# Metabolically Healthy and Unhealthy Obese Phenotypes among Arabs and South Asians: Prevalence and Relationship with Cardiometabolic Indicators

**DOI:** 10.3390/nu14050915

**Published:** 2022-02-22

**Authors:** Victor M. Oguoma, Mohamed Abu-Farha, Neil T. Coffee, Saad Alsharrah, Faisal H. Al-Refaei, Jehad Abubaker, Mark Daniel, Fahd Al-Mulla

**Affiliations:** 1 Faculty of Health, Health Research Institute, University of Canberra, Canberra 2617, Australia; neil.coffee@canberra.edu.au (N.T.C.); saad.alsharrah@dasmaninstitute.org (S.A.); mark.daniel@canberra.edu.au (M.D.); 2 Geohealth Laboratory, Dasman Diabetes Institute, Kuwait City 15462, Kuwait; faisal.alrefaei@dasmaninstitute.org; 3 Biochemistry and Molecular Biology Department, Dasman Diabetes Institute, Kuwait City 15462, Kuwait; mohamed.abufarha@dasmaninstitute.org (M.A.-F.); jehad.abubakr@dasmaninstitute.org (J.A.); 4 Department of Medicine, St. Vincent’s Hospital, The University of Melbourne, Melbourne 3010, Australia; 5 Genetics and Bioinformatics Department, Dasman Diabetes Institute, Kuwait City 15462, Kuwait; fahd.almulla@dasmaninstitute.org

**Keywords:** metabolically healthy obese, hs-CRP, ALT, HOMA-IR, Arabs, South Asian, Kuwait

## Abstract

Obesity is a public health crisis in Kuwait. However, not all obese individuals are metabolically unhealthy (MuHO) given the link between obesity and future cardiovascular events. We assessed the prevalence of the metabolically healthy obese (MHO) phenotype and its relationship with high sensitivity C-reactive protein (hs-CRP), serum alanine aminotransferase (ALT), and insulin resistance (HOMA-IR) in Arab and South Asian ethnic groups in Kuwait. The national cross-sectional survey of diabetes and obesity in Kuwait adults aged 18–60 years were analysed. The harmonised definition of metabolic syndrome was used to classify metabolic health. Multinomial logistic regression analysis was used to model the relationship between the MHO and MuHO phenotypes and hs-CRP, ALT and HOMA-IR levels. Overall, the prevalence of MHO for body mass index (BMI)- and waist circumference (WC)-defined obesity was 30.8% and 56.0%, respectively; it was greater in women (60.4% and 61.8%, respectively) than men (39.6% and 38.2%, respectively). Prevalence rates were also lower for South Asians than for Arabs. The MHO phenotype had hs-CRP values above 3 µg/mL for each age group category. Men compared to women, and South Asians compared to Arabs had a lower relative risk for the MHO group relative to the MuHO group. This study shows there is high prevalence of MHO in Kuwait.

## 1. Introduction

Global estimates indicate steady and highly concerning increases in the burden of overweight and obesity. In 2016, over 1.9 billion and 650 million adults above 18 years were overweight and obese, respectively [1]. Obese individuals are at an increased risk for cardio-metabolic complications than healthy-weight individuals [2]. Cardiometabolic complications linked with obesity include type 2 diabetes mellitus (T2DM), hypertension, dyslipidaemia, coronary heart disease, stroke, and some cancers. Sims [3] initially proposed the concept of the metabolically healthy obese phenotype in 2001. The relevance of maintaining a healthy body weight to reduce the risk of cardiometabolic complications is well understood amongst health scientists. However, the recognition that disease risks might vary among obese individuals has led to more inquiry to delineate different phenotypes. These subgroup of individuals without obesity-related risk factors are called “metabolically healthy obese” (MHO) [4].

Several classifications of the MHO phenotype have been suggested. The harmonised definition from the International Diabetes Federation refers to individuals without the conventional risk factors for metabolic syndrome, such as hyperglycaemia, dyslipidaemia (elevated blood triglyceride and lowered HDL-cholesterol), and hypertension [5,6]. Other definitions have considered the absence of insulin resistance and systemic inflammation [7,8]. The prevalence of MHO also varies based on the classification used.

Existing evidence has suggested a relationship between serum alanine aminotransferase (ALT), a marker of liver dysfunction and obesity in humans [9,10]. In clinical and population studies, ALT has been shown to predict T2DM and metabolic syndrome, given its relationship with insulin resistance [11,12]. Insulin resistance is an underlying factor of metabolic syndrome, which is predisposed by both genetic and acquired factors (such as dietary intake) [13,14]. High sensitivity C-reactive protein (hs-CRP), a marker of systemic inflammation and predictor of T2DM and cardiovascular disease (CVD), has been shown to associate with metabolic syndrome and its components [15,16]. However, what is yet to be known, especially in the context of the Kuwait population where the burden of obesity and CVD is high, is how these markers relate with the metabolically healthy and unhealthy obese phenotypes.

In Kuwait, a recent report in 2021 showed an obesity prevalence of 42.1% (95%CI: 40.0–44.3%) [17]. However, this prevalence rate did not delineate between MHO and metabolically unhealthy obese (MuHO) individuals. Studies have found that measures of central obesity, such as the waist circumference (WC), are superior to the body mass index (BMI) in predicting cardiometabolic risk [18,19]. Kuwait is known to have some of the highest rates of obesity globally. While efforts to mitigate the growing epidemic of obesity are underway, an understanding of the characteristics of the MHO and MuHO subgroups could potentially aid targeted obesity prevention and management interventions. The objectives of this study were: to determine the prevalence of the MHO phenotype in Arab and South Asian ethnic groups in Kuwait; to assess the differences between the phenotypes based on the definition of obesity (BMI vs. WC); and to assess the relationship between the MHO and MuHO, and hs-CRP, ALT, and insulin resistance, quantified using the homeostasis model assessment (HOMA).

## 2. Materials and Methods

### 2.1. Study Design and Participants

We studied 5291 subjects from the national population-based cross-sectional survey of diabetes and obesity in Kuwait, part of the Kuwait Diabetes Epidemiology Program conducted between 2011 to 2014 on Arab and South Asian adults aged 18–82 years. A stratified random sample of participants by nationality and governorate from the computerised register of the Public Authority for Civil Information (PACI) was used, as earlier reported [17]. A total of 1712 participants remained for analysis after the exclusion of records without hs-CRP measures. South Asian expatriates residing in Kuwait were predominantly from India, Pakistan, Sri Lanka, and Bangladesh; Arab groups were mainly from Kuwait, Egypt, Lebanon, Syria, Jordan, Iran, Palestine, and Yemen, as previously described [20]. Given that this is a secondary analysis of the data from the Kuwait Diabetes Epidemiology program, our prevalence estimates of MHO from this subsample (1712 participants) will be generalisable at 8% margin of error, and 90% confidence level, assuming a 24% baseline prevalence of MHO [21], and 40% non-response rate.

### 2.2. Anthropometric Measurements

Anthropometric measurements were carried out on participants in light clothing and barefoot. The procedure and measuring instrument used to evaluate obesity and hypertension in this study have been published in our previous study [17,20]. The body mass index (BMI) was used to classify participants into underweight (<18.5 kg/m^2^), normal weight (18.5–24.9 kg/m^2^), overweight (25.0–29.9 kg/m^2^), and obese (≥30 kg/m^2^). Waist circumference (WC) > 94 cm in Arab men and >80 cm in Arab women, and WC ≥ 90 cm for South Asian men and ≥80 cm for South Asian women were used to classify central obesity [22].

### 2.3. Biochemical Measurements

The method used for the measurement of biochemical parameters in this survey has been published previously [17,20]. For brevity, study participants fasted for at least 10 h prior to the collection of fasting blood samples. The Siemens Dimension RXL chemistry analyser (Diamond Diagnostics, Holliston, MA, USA) was used to measure the blood glucose and lipids profile. The participants were measured for HbA1c using a Variant device (Bio-Rad Laboratories, Hercules, GA, USA). The Access Ultrasensitive Insulin Assay (Beckman Coulter, Brea, CA, USA) was used in measuring insulin. Insulin resistance was calculated using the HOMA-IR formula: FBG (mmol/L) × fasting insulin (mU/L)/22.5 [23]. Hs-CRP concentrations were determined using ELISA (Hycult Biotech, Cat. no. HK369, Uden, The Netherlands), while ALT values in blood were analysed using the Dimension EXL Chemistry Analyser (Berlin, Germany) following manufacturer’s instructions. All blood analyses were conducted at the Dasman Diabetes Institute clinical laboratories in Kuwait.

### 2.4. Definition of Metabolically Healthy Obesity

The criteria for defining the metabolically healthy obese (MHO) phenotype is presented in Table 1. General obesity and central obesity are defined as BMI ≥ 30 kg/m^2^ with ethnic-specific WC thresholds applied as outlined in Section 2.2 above, respectively. Metabolically healthy status was defined as the presence of any two of the following: elevated blood pressure, elevated blood triglycerides, low HDL cholesterol, and hyperglycaemia. Based on the combination of obese categories (obese vs. normal weight) and metabolic health status (healthy vs. unhealthy), the participants were categorised into 4 groups: metabolically unhealthy normal weight [MuHN], metabolically unhealthy obese [MuHO], metabolically healthy normal weight [MHN], and metabolically healthy obese [MHO].

### 2.5. Ethical Clearance

This study was conducted at the Dasman Diabetes Institute and approved by the Ethical Review Committee (ERC-RA2010-004). The study protocol was consistent with the Declaration of Helsinki. All participants signed the consent form, in writing, prior to enrolment in the study.

### 2.6. Statistical Analysis

Descriptive statistics were used to describe the basic demographic characteristics and biochemical markers in the metabolically healthy (normal weight vs. obese) and unhealthy (normal weight vs. obese) subgroups of the study population. Two separate multinomial logistic regression models, one for each of the BMI- and WC-defined MHO and MuHO phenotypes, were used to assess the association of the unordered categorical response outcomes (MuHN, MuHO, MHN, and MHO) with the biochemical markers (hsCRP, ALT, and HOMA-IR), while accounting for age, gender, and ethnic group of the study participants. Model estimates are reported as relative risk ratio (RRR). The Hausman–McFadden test of independence of irrelevant alternatives (IIA) was used to assess the assumption that preference for a given choice of response level is unaffected by the presence of the ones not involved in the RRR estimation [24]. From the fitted models, we assessed the non-linear relationship of the outcome by gender and ethnic group for each of the covariates (hsCRP, ALT, and HOMA-IR). Statistical significance was set at *p* < 0.05. All statistical analyses and visualisations were conducted using Stata 17.0 (StataCorp, College Station, TX, USA).

## 3. Results

There were 1712 participants analysed in this study comprising of 907 Arabs and 805 South Asians with mean ages of 47 and 44 years, respectively. Table 2 shows the characteristics of participants based on BMI- and WC-defined obesity for the different phenotypes of metabolically healthy and unhealthy obesity. For BMI- and WC-defined obesity, women had a higher prevalence of MHO than men (60.4% and 61.8%, versus 39.6% and 38.2%, respectively). South Asians had a lower percentage of the MHO phenotype than Arabs for BMI- and WC-defined obesity: 33.5% and 37.9 versus 66.5% and 62.1%, respectively.

In both BMI- and WC-defined obesity, the MuHO phenotype had the highest mean and median ALT (45.3 and 43.6), AST (26.2 and 25.1), HS-CRP (4.7 and 3.8), and HOMA-IR (4.0 and 3.6), respectively, compared to the MHO, MHN, and MuHN groups. The MuHN phenotype had the highest mean serum creatinine values compared to the other phenotypes.

**Figure 1:**
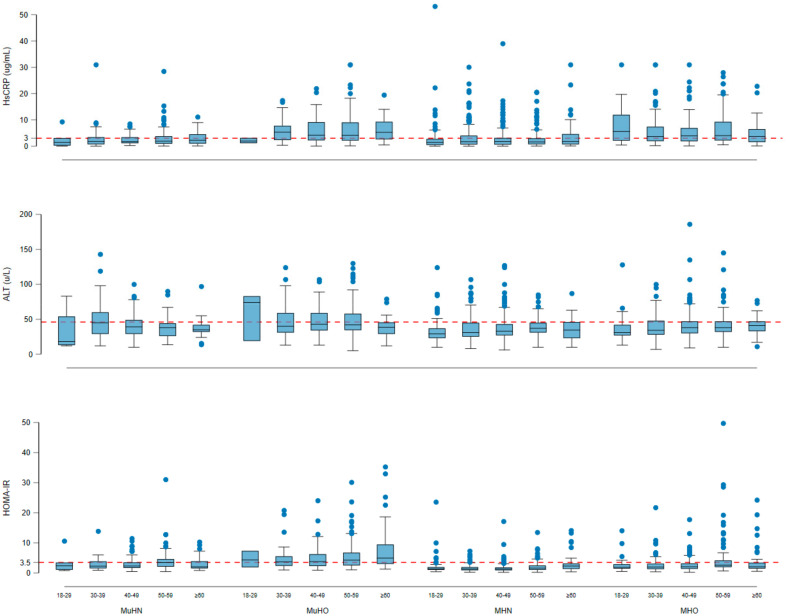
The dash red line for hs-CRP is the population cut-off point 3 µg/mL; ALT (46 µL) and HOMA-IR (3.5) is the 75th percentile of the population values. The distribution of hs-CRP, ALT, and HOMA-IR by age group and phenotypes of metabolically healthy/unhealthy obesity.

Figure 1 shows the distribution of hs-CRP, ALT, and HOMA-IR by age group and phenotypes of metabolically healthy and unhealthy obesity. The median hs-CRP value 5.6 µg/mL (25th–75th percentile: 2.1–11.9 µg/mL; *n* = 35) for MHO was the highest among younger adults 18–29 years old compared to older adults, while the median hs-RCP value for MuHO increased as the age category increased. For HOMA-IR, the MuHO group had the median values across age groups above the 75th percentile (3.5) of the population HOMA-IR value.

**Figure 2:**
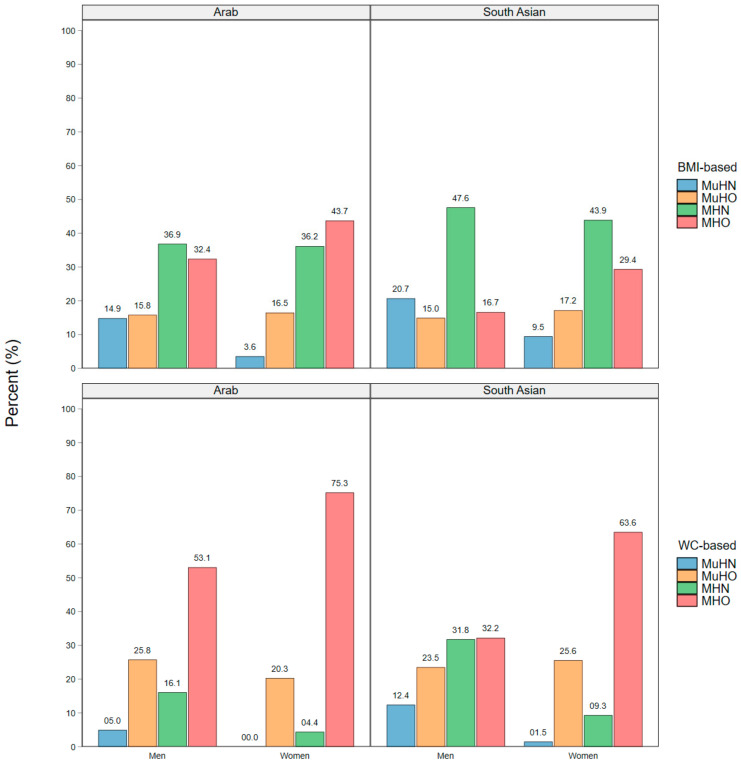
Prevalence of the BMI- and WC-defined metabolically healthy and unhealthy obese phenotypes by gender and ethnic group.

The prevalence of the MHO, MuHO, MHN, and MuHN phenotypes by ethnic group and gender is shown in Figure 2. In both genders, the prevalence of MHN was higher in the South Asian group compared to Arabs for the BMI-based obese classification, while there were proportionately more MHO Arab men and women compared to their South Asian counterparts. For the WC-defined obese classification, MHO prevalence was higher in women than men, but was highest in Arab women and men than their South Asian counterparts.

Table 3 shows the multinomial regressions analysis of the adjusted association between ALT, HOMA-IR, and hs-CRP, and the MHO, MuHO, MHN, and MuHN phenotypes. For the BMI-based obese classification, a unit increase in ALT (RRR = 0.97, 95%CI: 0.97–0.98, *p* < 0.001), HOMA-IR (RRR = 0.63, 95%CI: 0.58–0.69, *p* < 0.001), and hs-CRP (RRR = 0.90, 95%CI: 0.87–0.93, *p* < 0.001) values was associated with a lower relative risk of being in the MHN phenotype relative to the MuHO phenotype. Additionally, a unit increase in ALT, HOMA-IR, and hs-CRP values were associated with lower risk of being in the MuHN and MHO phenotype relative to the MuHO phenotype, except for hs-CRP in the MHO phenotype relative to the MuHO phenotype, where the association was not statistically significant (RRR = 0.98, 95%CI: 0.96–1.01, *p* = 0.20). For women compared to men, the relative risk for MHN relative to MHO would be expected to decrease by 41% (95% CI: 16–58%, *p* = 0.003), and decrease by 66% (95%CI: 48–78%, *p* < 0.001) for MuHN relative to MuHO in the adjusted analysis. For ethnic groups, South Asians compared to Arabs had a statistically significant lower relative risk for the MHO phenotype relative to the MuHO phenotype.

**Figure 3:**
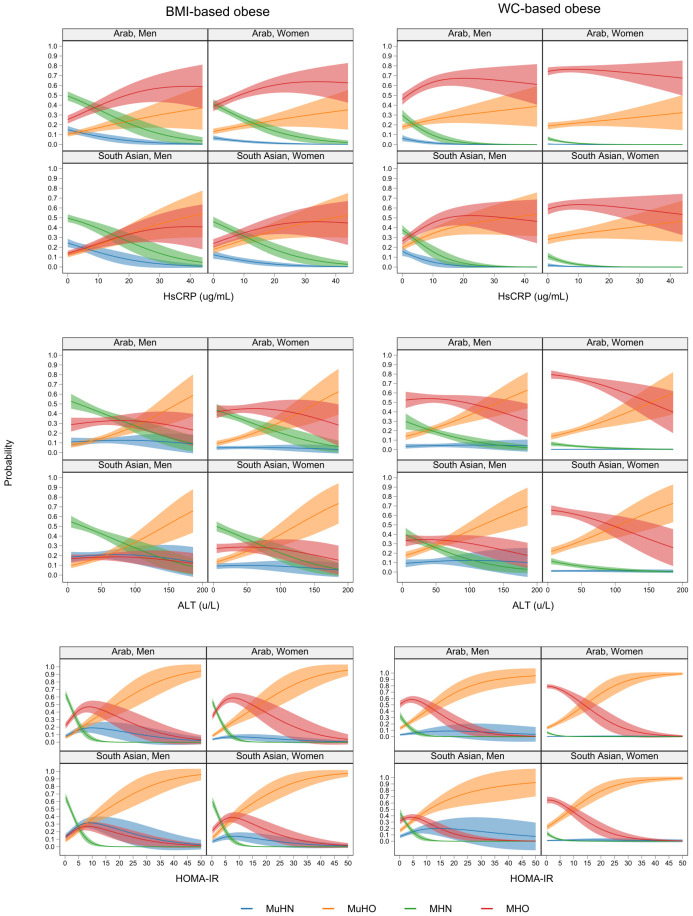
Non-linear relationship between hs-CRP, ALT, and HOMA-IR, and the metabolically healthy and unhealthy obese phenotypes by ethnic group and gender.

The non-linear relationship between hs-CRP, ALT, and HOMA-IR and the MHO, MuHO, MHN, and MuHN phenotypes are presented in Figure 3. For BMI-based obesity, the probability of MHO was highest, followed by MuHO, and increased for every unit increase in the hs-CRP value, especially for the Arab group. The trend was similar in the South Asian group. However, there was no difference between the MHO and MuHO phenotypes. The probability of the MuHN and MHN phenotypes for both gender and ethnic groups declined for every unit increase in the hs-CRP values. For WC-based obesity, the probability of MHO was the highest, in which it increased, plateaued at about 10 µg/mL, then decreased gradually following a unit increase in the hs-CRP value. Unlike the MHO phenotype, the probability of the MuHO phenotypes rose steadily with an increased hs-CRP value for both gender and ethnic groups.

The trend in the probability of the MHO and MuHO phenotypes for ALT and HOMA-IR showed that across anthropometric-defined obesity, gender, and ethnic categories, only the MuHO phenotypes increased steadily following a unit increase in the ALT value. For every unit increase in the hs-CRP, ALT, and HOMA-IR values, the probability of MHN decreased.

## 4. Discussion

In this sample of 1712 Kuwaiti Arabs, Arabs from Middle Eastern and Mediterranean countries, and expatriates from South Asia, the overall prevalence of MHO based on the consensus definition and according to BMI- and WC-defined obesity was 30.8% (95%CI: 28.7 to 33.1%) and 56.0% (95%CI: 52.9 to 57.7%), respectively. The MHO phenotype across each age group category had hs-CRP values above 3 µg/mL. This shows that high hs-CRP value is an important cardiometabolic syndrome component and should be considered in the screening of cardiometabolic syndrome in Kuwait population, especially among the younger population given that the age group 18–29 years had the highest median hs-CRP value.

In both BMI- and WC-defined obesity, our study found that the MuHO phenotype has the highest mean or median hs-CRP, ALT, and HOMA-IR values compared to other phenotypes. However, the relationship between these markers and MHO are of particular interest given the high prevalence of diabetes, obesity, and associated increased CVD risk in the Kuwait population [17,20,25,26], and the understanding that obesity is not invariably associated with adverse metabolic conditions [27].

For both genders, the prevalence of MHN was higher in the South Asian group compared to Arabs for BMI- and WC-based obese classifications, while there were more MHO Arab men and women compared to their South Asian counterparts. Previous research that compared obesity and metabolic risk factors of atherosclerosis in Arabs and South Asians found that Arabs were not only more obese, but also developed obesity at a younger age than South Asians [28]. The delineation of metabolically healthy/unhealthy and normal/obese in our study demonstrates a much greater burden of obesity among metabolically healthy Arabs relative to South Asian counterparts in Kuwait. A recent study that assessed the natural courses of different phenotypes and their associations with CVD risks in China found that one-third of MHO individuals became unhealthy within 5 years of their initial assessment, and that MHO individuals compared to MHN counterparts had higher odds of increased brachial-ankle pulse wave velocity (baPWV), a measure of arterial stiffness, incident hypertension, and diabetes [29]. This suggests that the absence of CVD risk factors in obese individuals does not attenuate the positive association between obesity and cardiovascular mortality.

For WC-defined obesity, the MHO prevalence was higher in women than men, and highest in women and men Arabs compared to their South Asian counterparts. The gender and ethnic category differences in the prevalence of MHO underscore the higher burden of obesity in the Arab ethnic group compared to the South Asian group, and differences in body fat distribution between gender. Existing evidence in the United States of America reports that women typically present with about 10% higher body fat compared to men [30]. Higher rates of central obesity are very commonly reported in women than in men due to biological and social determinants of obesity, such as genetic factors, physical activity level, socio-cultural beliefs, and urbanisation [31,32]. For example, our recent report from the data from this population showed that women had higher insufficient physical activity levels compared to men [33].

Apart from the consensus classification of the metabolic and obesity phenotypes utilised in this study, others have found strong association of systemic inflammation (measured by hs-CRP) and insulin resistance (HOMA-IR) in the development of metabolic syndrome [7]. ALT has also been implicated to be strongly associated with liver dysfunction and obesity [9,10]. Our findings on the relationship between the MHO and MuHO phenotypes with hs-CRP, ALT, and HOMA-IR show that for the BMI-based obese group, the probability of being in the MHO group was higher compared to the MuHO group, and increased per unit for every increase in the hs-CRP value, especially for the Arab group. This suggests that in our study population, obesity was a stronger driver of systemic inflammation, especially in the Arab group than in the South Asian group. Metabolically healthy obese individuals were perhaps at an early stage of metabolic transition driven by abnormal or excessive fat accumulation, which may trigger a high release of inflammatory mediators (acute inflammation) [34]. As a risk factor, inflammation is an entrenched mechanism for developing CVD, including coagulation, atherosclerosis, metabolic syndrome, insulin resistance, and diabetes mellitus, as well as non-CVDs (psoriasis, depression, cancer, and renal diseases) [35]. The higher probability of MHO compared to MuHO as the hs-CRP level increases may result from the saturation of immune responses in MuHO individuals, which are at a more chronic stage.

The probability of MHO declined with increases in the ALT values in both gender and ethnic groups, especially for the WC-based obese group. There was a consistent increase in the probability of MuHO, which highlights the potential liver injury attributable to the contribution of cardiometabolic conditions and obesity in both ethnic and gender groups. A recent study demonstrated a relationship between serum ALT and obesity but, unfortunately, the mechanism(s) was not clearly elucidated [9]. A population-based study in Korea showed higher odds of elevated ALT for a given increase in BMI with odds ratio of 5.0 and 3.9 for elevated ALT in obese men and women, respectively [36]. Another study in the United States of America that assessed the relationship between ALT and body fat showed that central obesity was the major body composition determinant of increased ALT, thereby supporting the hypothesis that liver injury can be induced by metabolically active intra-abdominal fat [37].

Trends in the probability of the MHO and MuHO phenotypes for HOMA-IR show that across anthropometric-defined obese, gender, and ethnic categories, only the MuHO phenotype increased steadily following a unit increase in the HOMA-IR value. This finding aligns with studies reporting strong positive associations between metabolic syndrome and HOMA-IR [38,39]. In the current study, for BMI-defined obesity in the Arab ethnic group, the probability of being in the MHO group rose to the 90th percentile value (~6) but then dropped as the HOMA-IR values increased. Similar trends were apparent for the South Asian group but at a lower probability. Differences in non-linear relationships between the WC- and BMI-defined obesity groups and phenotypes can be accounted for by the differences in body fat expression represented by the different anthropometric indices used to classify obesity. The processes that influence the different effects of obesity on metabolic health are not clear, however, studies carried out in rodent models suggest differences in adipose tissue morphology related to weight gain can cause or prevent systemic metabolic dysfunction [40]. Thus, leading to a small subgroup of individuals with obesity having normal HOMA-IR and no metabolic syndrome component.

The clinical relevance in the stratification of obese individuals with respect to their metabolic health phenotype is results that advance the basis and need for the early identification of individuals to be prioritised for pharmacological and lifestyle intervention [41]. This is pertinent as obesity treatment guidelines do not differentiate between MHO and MuHO individuals [42]. In populations such as Kuwait where obesity is highly prevalent among younger adults [17], early intervention becomes imperative, especially given that our study found a higher hs-CRP value in 18–29-year-old individuals with MHO compared to those with MuHO. Therefore, prioritisation of targeted public awareness campaigns, community outreach programs, and mass media campaigns designed to enlighten the Kuwait populace and promote healthier food choices and lifestyles in the prevention of obesity and associated CVD risks, especially among the high-risk groups identified here, will help in taming the substantial burden of obesity and cardiometabolic syndrome in Kuwait.

### Strengths and Limitations of the Study

All parameters for classifying the metabolic status of the study participants were objectively measured, supporting the validity of our measures. Additionally, our gender and ethnic group comparisons are unique and have not been previously performed for Kuwait. Data used in this study are from a cross-sectional design, which precludes knowledge of temporality and causality in the associations explored. Duration of cardiometabolic risk factors was not accounted for, so our analysis was unable to adjust for length of disease among the study participants. Given that parameter estimates were not weighted to the overall population of Kuwait, our findings are not nationally representative, but applicable to the sample analysed.

## 5. Conclusions

This study demonstrates a higher proportion of MHO in our analytic sample in Kuwait. MHO was higher among women than men, and higher among Arabs than South Asians living in Kuwait. The high hs-CRP value among MHO individuals indicates that hs-CRP is an important cardiometabolic syndrome component and should be considered in the screening of cardiometabolic syndrome in the Kuwait population, especially among the younger population. A high probability of inflammation among MHO individuals suggests that the absence of CVD risk factors in obese individuals is unlikely to attenuate the known, positive association between obesity and cardiovascular mortality. Given that >30% of the sample population are MHO, prioritising holistic population-based and multilevel approaches to reduce the burden of obesity in Kuwait will lead to better health outcomes.

## Figures and Tables

**Table 1:** Criteria for defining the MHO phenotypes.

S/No		Definition [5,22]	
		BMI-Based	WC-Based
1	Blood pressure	Systolic/diastolic blood pressure ≥ 130/85 mmHg or receiving treatment for hypertension	Systolic/diastolic blood pressure ≥ 130/85 mmHg or receiving treatment for hypertension
2	Blood triglyceride	≥1.7 mmol/L	≥1.7 mmol/L
3	HDL cholesterol	<1.0mmol/L in men or <1.3 mmol/L in women	<1.0mmol/L in men or <1.3 mmol/L in women
4	Elevated blood glucose	≥5.6 mmol/L or receiving medication	≥5.6 mmol/L or receiving medication
5	Elevated waist circumference or BMI	BMI ≥ 30 kg/m^2^	Ethnic-specific WC thresholds ^†^

^†^ WC cut-point for Europid ethnicity was used to define central obesity for Arabs (men WC ≥ 94 cm and women WC ≥ 80 cm), and ethnic-specific cut-off for South Asians (men WC ≥ 90 cm and women ≥ 80 cm), S/No = serial number.

**Table 2:** Sample characteristics for the BMI- and WC-defined metabolically healthy and unhealthy obese phenotypes.

	BMI-Based Obesity					WC-Based Obesity				
	MuHN	MuHO	MHN	MHO	*p*-Value	MuHN	MuHO	MHN	MHO	*p*-Value
	N = 207	N = 275	N = 702	N = 528		N = 82	N = 398	N = 264	N = 947	
Age (years)	48.8 (10.9)	50.8 (9.8)	42.5 (11.3)	45.8 (10.9)	<0.001	45.9 (11.6)	50.7 (9.9)	40.5 (10.8)	44.9 (11.1)	<0.001
Age group										
18–29	6 (2.9)	3 (1.1)	83 (11.8)	35 (6.6)	<0.001	3 (3.7)	6 (1.5)	38 (14.4)	78 (8.2)	<0.001
30–39	39 (18.8)	39 (14.2)	213 (30.3)	112 (21.2)		26 (31.7)	52 (13.1)	93 (35.2)	224 (23.7)	
40–49	62 (30.0)	73 (26.5)	224 (31.9)	198 (37.5)		22 (26.8)	113 (28.4)	81 (30.7)	337 (35.6)	
50–59	65 (31.4)	104 (37.8)	131 (18.7)	118 (22.3)		19 (23.2)	149 (37.4)	37 (14.0)	209 (22.1)	
≥60	35 (16.9)	56 (20.4)	51 (7.3)	65 (12.3)		12 (14.6)	78 (19.6)	15 (5.7)	99 (10.5)	
Gender										
Men	157 (75.8)	134 (48.7)	372 (53.0)	209 (39.6)	<0.001	77 (93.9)	212 (53.3)	211 (79.9)	362 (38.2)	<0.001
Women	50 (24.2)	141 (51.3)	330 (47.0)	319 (60.4)		5 (6.1)	186 (46.7)	53 (20.1)	585 (61.8)	
Ethnic group										
Arab	78 (37.7)	147 (53.5)	331 (47.2)	351 (66.5)	<0.001	20 (24.4)	205 (51.5)	87 (33.0)	588 (62.1)	<0.001
South Asian	129 (62.3)	128 (46.5)	371 (52.8)	177 (33.5)		62 (75.6)	193 (48.5)	177 (67.0)	359 (37.9)	
BMI (kg/m^2^)	26.4 (2.2)	35.7 (4.7)	26.0 (2.6)	35.1 (4.7)	<0.001	25.4 (3.0)	33.0 (5.6)	24.7 (2.9)	31.4 (5.6)	<0.001
WC (cm)	92.6 (7.0)	109.7 (11.1)	89.8 (9.3)	105.2 (12.3)	<0.001	87.7 (4.8)	105.3 (11.8)	83.6 (8.7)	100.0 (11.9)	<0.001
WHtR	0.6 (0.0)	0.7 (0.1)	0.5 (0.1)	0.6 (0.1)	<0.001	0.5 (0.0)	0.6 (0.1)	0.5 (0.0)	0.6 (0.1)	<0.001
TSH (uIc/mL)	2.3 (6.0)	2.4 (6.1)	2.3 (7.0)	2.0 (3.8)	0.78	1.8 (1.2)	2.4 (6.6)	2.5 (8.3)	2.1 (4.9)	0.59
FT3 (pmol/L)	4.9 (0.6)	4.8 (0.6)	4.9 (1.5)	4.8 (0.9)	0.10	4.9 (0.5)	4.8 (0.6)	5.0 (0.8)	4.9 (1.4)	0.27
ALT (u/L)	41.0 (20.3)	45.3 (21.4)	35.9 (17.3)	40.1 (18.5)	<0.001	43.1 (22.4)	43.6 (20.8)	36.4 (19.7)	38.2 (17.4)	<0.001
AST (u/L)	23.2 (10.9)	26.2 (13.7)	21.9 (11.0)	22.5 (10.8)	<0.001	24.1 (14.9)	25.1 (12.2)	22.8 (8.4)	22.0 (11.6)	<0.001
Creatinine (umol/L)	84.0 (20.4)	79.1 (30.1)	75.1 (18.5)	73.2 (17.2)	<0.001	87.2 (18.3)	79.2 (22.9)	81.6 (18.8)	72.5 (17.2)	<0.001
Hs-CRP (µg/mL)	1.8 (1.0–3.6)	4.7 (2.2–8.8)	1.7 (0.6–3.3)	3.9 (1.9–7.8)	<0.001	1.3 (0.8–2.8)	3.8 (1.8–7.5)	1.1 (0.4–2.4)	2.8 (1.2–6.0)	<0.001
HOMA-IR	2.4 (1.7–4.2)	4.0 (2.5–6.8)	1.4 (1.0–2.1)	2.2 (1.5–3.4)	<0.001	2.5 (1.5–4.6)	3.6 (2.1–6.0)	1.4 (0.9–2.1)	1.8 (1.2–2.9)	<0.001

BMI = body mass index, WC = waist circumference, WHtR = waist-to-height ratio, TSH = thyroid stimulating hormone, FT3 = Free triiodothyronine, ALT = serum alanine aminotransferase, AST = Aspartate aminotransferase, Hs-CRP = High sensitivity C-reactive protein, HOMA-IR = Homeostatic model assessment for insulin resistance, MuHO = metabolically unhealthy obese, MHN = metabolically healthy normal weight, MHO = metabolically healthy obese, MuHN = metabolically unhealthy normal weight.

**Table 3:** Association between hs-CRP, ALT, and HOMA-IR, and the metabolically healthy and unhealthy obese phenotypes.

BMI-Based Obese	MuHO (Reference Group)	MHN		MHO		MuHN	
	RRR (95% CI)	RRR (95% CI)	*p*-Value	RRR (95% CI)	*p*-Value	RRR (95% CI)	*p*-Value
Hs-CRP (µg/mL)	1.00 (1.00, 1.00)	0.90 (0.87, 0.93)	<0.001	0.98 (0.96, 1.01)	0.200	0.89 (0.84, 0.94)	<0.001
ALT (u/L)	1.00 (1.00, 1.00)	0.97 (0.97, 0.98)	<0.001	0.99 (0.98, 0.99)	<0.001	0.99 (0.98, 1.00)	0.004
HOMA-IR	1.00 (1.00, 1.00)	0.63 (0.58, 0.69)	<0.001	0.91 (0.87, 0.94)	<0.001	0.92 (0.87, 0.96)	0.001
Age	1.00 (1.00, 1.00)	0.94 (0.93, 0.96)	<0.001	0.96 (0.95, 0.98)	<0.001	0.99 (0.97, 1.01)	0.160
Gender							
Men	1.00 (1.00, 1.00)	1.00 (1.00, 1.00)	.	1.00 (1.00, 1.00)	.	1.00 (1.00, 1.00)	.
Women	1.00 (1.00, 1.00)	0.59 (0.42, 0.84)	0.003	1.13 (0.81, 1.58)	0.486	0.34 (0.22, 0.52)	<0.001
Ethnic group							
Arab	1.00 (1.00, 1.00)	1.00 (1.00, 1.00)	.	1.00 (1.00, 1.00)	.	1.00 (1.00, 1.00)	.
South Asian	1.00 (1.00, 1.00)	0.80 (0.57, 1.13)	0.204	0.44 (0.32, 0.62)	<0.001	1.34 (0.89, 2.01)	0.167
WC-based obese							
Hs-CRP (µg/mL)	1.00 (1.00, 1.00)	0.84 (0.78, 0.89)	<0.001	0.98 (0.96, 1.01)	0.159	0.84 (0.75, 0.93)	0.001
ALT (u/L)	1.00 (1.00, 1.00)	0.97 (0.96, 0.98)	<0.001	0.99 (0.98, 0.99)	<0.001	0.99 (0.98, 1.00)	0.131
HOMA-IR	1.00 (1.00, 1.00)	0.64 (0.56, 0.72)	<0.001	0.87 (0.84, 0.91)	<0.001	0.96 (0.89, 1.03)	0.212
Age	1.00 (1.00, 1.00)	0.91 (0.90, 0.93)	<0.001	0.96 (0.95, 0.97)	<0.001	0.96 (0.94, 0.98)	0.001
Gender							
Men	1.00 (1.00, 1.00)	1.00 (1.00, 1.00)	.	1.00 (1.00, 1.00)	.	1.00 (1.00, 1.00)	.
Women	1.00 (1.00, 1.00)	0.15 (0.10, 0.24)	<0.001	1.37 (1.04, 1.81)	0.023	0.08 (0.03, 0.21)	<0.001
Ethnic group							
Arab	1.00 (1.00, 1.00)	1.00 (1.00, 1.00)	.	1.00 (1.00, 1.00)	.	1.00 (1.00, 1.00)	.
South Asian	1.00 (1.00, 1.00)	1.17 (0.79, 1.73)	0.439	0.51 (0.39, 0.67)	<0.001	2.19 (1.22, 3.92)	0.01

BMI = body mass index, WC = waist circumference, Hs-CRP = High sensitivity C-reactive protein, ALT = serum alanine aminotransferase, HOMA-IR = Homeostatic model assessment for insulin resistance, MuHO = metabolically unhealthy obese, MHN = metabolically healthy normal weight, MHO = metabolically healthy obese, MuHN = metabolically unhealthy normal weight.

## Data Availability

The raw dataset analysed in this study is not publicly available. It can be provided on reasonable request to the Dasman Diabetes Institute through the corresponding author (V.M.O) and in line with the provisions of the ethics committee.
